# Cases and Dissections

**Published:** 1819-04-01

**Authors:** David Davies

**Affiliations:** Member of the Royal College of Physicians, and of the College of Surgeons in London; Senior Surgeon to St. Peter's Hospital, Bristol


### CASES and DISSECTIONS.

By David Davies, M. D. Member of the Royal College of Physicians, and of the College of Surgeons in London; Senior Surgeon to St. Peter's Hospital, Bristol.

CASE I. Morbid appearances of some of the abdominal and thoracic viscera of the body of Ann Collins, on examination, ten hours after death.

The omentum consisted of cellular substance only, although the subject externally had no deficiency of adeps, which was a little œdematous.

The urinary bladder was large, adhered extensively to the peritonæum, and contained a quart of urine.

The uterus was rather smaller than usual, for a woman who had borne children. The gall-bladder was much distended with bile. These excepted, neither the liver, kidnies, or any other of the abdominal viscera, bore mark of disease.

The lungs on the right side of the thorax were healthy, but on the left side, the superior anterior part was affected with tubercles in a state of suppuration, and the two lobes adhered strongly to each other; and on the anterior part of the lower lobe there had been inflammation which had terminated in gangrene, above which was an abscess, containing more than a table spoonful of a fluid of a blackish green colour.

Twelve ounces of fluid, tinged with blood, were contained in the pericardium, and that part of it contiguous to the abscess in the lungs had been inflamed.

The surface of the right and left ventricle, and of the left auricle of the heart, was covered with a layer of coagulable lymph, thicker than a shilling, and the right auricle was beautifully reticulated with the same matter. The left ventricle was inflamed, and adhered by means of coagulable lymph in one spot to the pericardium.

The heart measured in length, four inches. The diameter, at the base, was three inches and a half; and it weighed five ounces three-quarters, averdupoise. On examining the cavity of the head, it was observable, that the brain, its meninges, and ventricles were natural, but that the sinuses were loaded with blood.

The following history of the case was learnt from the friends of the deceased. Ann Collins was a married woman, twenty-nine years of age, and had borne four children: she had looked ill for the last twelve months, had expected to be unwell a week before Christmas 1810, but her catamenia disappeared from this time, though she had previously been regular. In April following, she was affected with a short cough, complained of pain in her left side, of numbness, which extended from her head to her toes; her eyes were sometimes affected with dimness; and on attempting to go up stairs one day she fainted, remained insensible for some time and incapable of moving, when assistance was procured to place her on a bed; she fainted once also whilst employed at needle-work.

Her abdomen was much enlarged; a medical man was now had to attend her, who supposed her pregnant, though she assured him that she was not so. No more than a quarter of a pint of urine was passed in the spacc of eleven days: her bowels were constipated; some purging medicines which she took, at length procured stools, and although the enlargement of the abdomen continued, she became gradually less in pain, and passed a small quantity of urine daily.

I shall subjoin the substance of the Reports which I received from her medical attendants, extracted from the minutes of the Registers of the charitable institutions where she had applied for relief.

Mr. Jenkins, Apothecary to the Bristol Dispensary, states, that Ann Collins was attended by him, in May 1811, that she had a swelling of the abdomen resembling pregnancy, but attended with great pain, and she positively denied being pregnant. He continued his attendance for six weeks, and although the swelling of the abdomen did not subside, she became gradually less in pain, and appeared better.

Mr. Beddingfield, Apothecary to the Bristol Infirmary states, that Ann Collins was admitted an in-patient into that house, on the 27th of June, 1811, that she was unable to support herself, and was led into the admission room by two women; her spirits were very much depressed, and her mind in a very anxious state.

She was much under the influence of mercury, and said that she had the dropsy; but he observing, that the tumour of the abdomen was more circumscribed than it usually is in this disease, he was led to conclude her pregnant; she passed her urine regularly, and in considerable quantities; but he adds in a note, that he found the urine come from her sometimes involuntarily. Her pulse was regular, and ninety strokes in a minute. He was confident that there was not present, at that time, any symptom which indicated a disease of the heart. She was then seen by the surgeon of the week, and dismissed as pregnant, on the 2d of July, 1811. The same day she was admitted, as apothecary's patient, into St. Peter's Hospital, where she was directed to take soluble tartar in small doses. Mr. Baker, the apothecary, did not think her in danger, her pulse being regular, and not exceeding ninety-six strokes in a minute.

The nurse of the ward being called to her at three o'clock in the morning, July the 8th, asked her if she thought her labour was coming on? To which she answered peevishly, how can it be my labour in my heart? She died at four o'clock the same morning, when more than three quarts of urine gushed from her, and the enlargement of the abdomen subsided.

Ann Collins having twice fainted, and the state of anxiety in which Mr. Beddingfield has described her to be when admitted into the Infirmary, induce a belief, that the effusion of a fluid into the pericardium had occurred previous to the peritoneal inflammation and the retention of urine, for which Mr. Jenkins had attended this patient.

During the six days she remained in St. Peter's Hospital, we learn, that no symptom of serious disease was discovered by a very intelligent and ingenious medical practitioner, Mr. Baker.

Here, however, independent of a partial retention of urine forming a circumscribed tumour of the abdomen, is an instance of the heart having suffered organic lesion, the lungs inflammation, abscess, and gangrene, the pericardium containing an effusion of fluid amounting to twelve ounces, and yet the usual symptoms of mischief remaining obscure.

Mr. Beddingfield, in his Compendium of Medical Practice of the Bristol Infirmary, has thus alluded to the Case of Ann Collins. Chap. II. page 137.

"I am not acquainted with the minutiæ of the dissection, neither have I been very anxious to obtain them, as a gentleman in this city has it in contemplation to publish the case, with a plate representing some ulcerations which existed upon the surface of the heart." And in page 138, Mr. B. observes, "Perhaps a greater number of circumstances scarcely ever concurred in any one case to deceive medical men as to the real condition of the sanguiferous, uterine, and urinary systems." *In loco citato.*

CASE 2. Thomas Sinnot, 76 years of age, by trade a Taylor, had formerly complained once of vertigo. On Saturday, the 4th of May, 1816, he felt quite well, and having worked in the forenoon at his trade, afterwards walked to the house of a friend, intending to take his dinner with him, but *before* dinner, Sinnot expired suddenly.

On the following day I examined the body of the deceased, for the purpose of ascertaining the cause of death. The heart, lungs, and arteries were found perfectly free from all appearance of disease. The contents of the abdomen appeared also sound.

*Examination of the Head*. On removing the calvaria, the dura mater was found to adhere firmly, the sinuses were not over distended with blood, but the ventricles of the brain were filled with a serous limpid fluid; the medulla oblongata was small and firm; the brain in other respects appeared in a natural state.

Does not the case of Thomas Sinnot present an instance of serous apoplexy terminating suddenly fatal, where little or no previous indisposition had been manifest, and where the state of the thoracic and abdominal viscera might have served the purpose of existence many years? Or should this case be considered as an example of hydrocephalus internus?

CASE 3. On the 17th of September, 1818, a lad, 11 years of age, shewed slight symptoms of fever, for which a few grains of pulvis antimonalis were given every four hours, and leeches were directed to be applied to the temples. He was not considered to be in danger either by the physician or apothecary who attended him, but he expired early on the morning of the 27th, and was reported by his physician "to have died suddenly of fever."

Being requested to examine the body, thirty-six hours after death, the external appearance of the abdominal region induced me to suggest that the lad had died of peritoneal inflammation. The thoracic vicera were healthy. The abdomen was distended with a turbid fluid; the peritoneum and peritoneal covering of the ileum pourtrayed such decided marks of inflammation and gangrene, that no question could remain of the cause of death in this subject. A small portion only of the internal surface of the ileum exhibited any appearance of the late inflammation.

Such events should impress our minds with the importance of a minute examination of the symptoms which present themselves in all cases of pyrexia; for I think it cannot be doubted that the fever here was excited by local inflammation, and could have been relieved by active measures only, pursued with a view to diminish it. I abstain from further remarks, trusting that the bare relation of the facts may induce some of your scientific correspondents to enlarge on the subject, and enforce the utility of post mortem examinations.

